# Incidence of Type 2 Diabetes in Japan: A Systematic Review and Meta-Analysis

**DOI:** 10.1371/journal.pone.0074699

**Published:** 2013-09-06

**Authors:** Atsushi Goto, Maki Goto, Mitsuhiko Noda, Shoichiro Tsugane

**Affiliations:** 1 Department of Diabetes Research, Diabetes Research Center, National Center for Global Health and Medicine, Tokyo, Japan; 2 Epidemiology and Prevention Division, Research Center for Cancer Prevention and Screening, National Cancer Centre, Tokyo, Japan; National Taiwan University, Taiwan

## Abstract

**Background:**

The definition of incident type 2 diabetes varies across studies; hence, the actual incidence of type 2 diabetes in Japan is unclear. Here, we reviewed the various definitions of incident type 2 diabetes used in previous epidemiologic studies and estimated the diabetes incidence rate in Japan.

**Methods:**

We searched for related literature in the MEDLINE, EMBASE, and *Ichushi* databases through September 2012. Two reviewers selected studies that evaluated incident type 2 diabetes in the Japanese population.

**Results:**

From 1824 relevant articles, we included 33 studies with 386,803 participants. The follow-up period ranged from 2.3 to 14 years and the studies were initiated between 1980 and 2003. The random-effects model indicated that the pooled incidence rate of diabetes was 8.8 (95% confidence interval, 7.4–10.4) per 1000 person-years. We observed a high degree of heterogeneity in the results (I^2^ = 99.2%; p < 0.001), with incidence rates ranging from 2.3 to 52.6 per 1000 person-years. Three studies based their definition of incident type 2 diabetes on self-reports only, 10 on laboratory data only, and 20 on self-reports and laboratory data. Compared with studies defining diabetes using laboratory data (n = 30; pooled incidence rate = 9.6; 95% confidence interval = 8.3–11.1), studies based on self-reports alone tended to show a lower incidence rate (n = 3; pooled incidence rate = 4.0; 95% confidence interval = 3.2–5.0; p for interaction < 0.001). However, stratified analyses could not entirely explain the heterogeneity in the results.

**Conclusions:**

Our systematic review and meta-analysis indicated the presence of a high degree of heterogeneity, which suggests that there is a considerable amount of uncertainty regarding the incidence of type 2 diabetes in Japan. They also suggested that laboratory data may be important for the accurate estimation of the incidence of type 2 diabetes.

## Introduction

The prevalence of type 2 diabetes is increasing globally and the International Diabetes Federation has predicted that the number of people with diabetes will increase from 366 million to 552 million by 2030 [1]. Importantly, the prevalence of diabetes in Asia is rapidly increasing as 60% of the world’s diabetic population are Asians [2]. In Japan, the estimated number of individuals with diabetes was approximately 6.9 million in 1997 [3], 7.4 million in 2002 [4], and 8.9 million in 2007 [5]. Although the estimates of the prevalence of diabetes have been computed from the National Health and Nutrition Survey of Japan, the incidence rate of type 2 diabetes in Japan has not been fully clarified. Furthermore, the definition of incident type 2 diabetes varies across studies. Changes in the diagnostic criteria for diabetes may account for these discrepancies [6–8]. The American Diabetes Association (ADA), World Health Organization (WHO), and Japan Diabetes Society (JDS) lowered the fasting plasma glucose (FPG) threshold from 140 to 126 mg/dL in 1997, 1998, and 1999, respectively [6,8,9]. In 2009, an International Expert Committee recommended the use of HbA1c level (with a threshold of ≥6.5% (48 mmol/mol) [10]) to diagnose diabetes, and the ADA, WHO, and JDS adopted this criterion in 2010, 2011, and 2010, respectively [11–13]. However, in epidemiologic studies, measuring HbA1c or blood glucose is sometimes difficult for various reasons such as inconvenience or high costs. Therefore, several studies used self-reported diabetes as an outcome if laboratory findings were not available and self-administered questionnaires concerning diabetes history were [14,15]. However, the definition of diabetes diagnosis in epidemiologic studies remains controversial. Therefore, we conducted this systematic review and meta-analysis to estimate the incidence rate of type 2 diabetes in Japan and compile the various definitions of incident type 2 diabetes used in previous epidemiologic studies.

## Methods

### Search Strategy

This systematic review and meta-analysis did not have a registered review protocol, but was performed according to the recommendations of the Preferred Reporting Items for Systematic Reviews and Meta-Analyses (PRISMA) Group [16]. We searched the MEDLINE, EMBASE, and *Ichushi* (*Japana Centra Revuo Medicina*) databases through September 2012. Two reviewers selected studies that evaluated newly diagnosed type 2 diabetes among the Japanese population. The MEDLINE search terms were ("diabetes mellitus, type 2"[MeSH Terms] OR "type 2 diabetes mellitus"[All Fields] OR "type 2 diabetes"[All Fields]) AND ("risk"[MeSH Terms] OR "risk"[All Fields] OR "incidence"[MeSH Terms] OR "incidence"[All Fields]) AND ("Japan"[MeSH Terms] OR "Japan"[All Fields]). Similar search terms were used for searching the EMBASE and *Ichushi* databases. We further searched the references of relevant studies.

### Selection

Two independent reviewers read all the retrieved abstracts and titles. The predefined inclusion criteria were as follows: 1) new-onset of type 2 diabetes reported as a study outcome and 2) study on the Japanese population. The full text of studies meeting these criteria was retrieved and screened to determine eligibility, and studies on the same participant groups were excluded. Discrepancies between the reviewers’ selection were resolved by discussion.

### Data Extraction

The information extracted by 2 investigators (AG and MG) was as follows: study characteristics (authors, design, year of publication, year(s) when the studies were conducted, sample size, and duration of follow-up), participants’ characteristics (age and gender), outcome assessment (definition of incident diabetes), analysis strategy, and validity studies (sensitivity, specificity, positive predictive value, and negative predictive value). HbA1c values are presented in percentage units as per the National Glycohemoglobin Standardization Program (NGSP) and in the units (mmol/mol) recommended by International Federation of Clinical Chemistry and Laboratory Medicine (IFCC) [17].

### Data Synthesis

In studies with sufficient information on incident type 2 diabetes, we calculated the incidence rate per 1,000 person-years by dividing the number of incident diabetes cases by the duration of follow-up. When the mean follow-up duration was not available, the median was used. We used exact methods based on the Poisson distribution to compute the 95% confidence interval (CI) for each study [18]. The incidence rates of included studies were pooled on the log scale using inverse variance weighting and the random-effects model to calculate a pooled diabetes incidence rate and 95% CIs [19]. We assessed statistical heterogeneity of incidence rates across studies using the Cochrane’s Q test [20] and I^2^ statistic [21]. Potential publication bias was assessed using funnel plots, Begg’s test [22], and Egger’s test [23]. We also performed stratified analyses according to the definition of incident diabetes (self-report vs. laboratory data), source of subjects (population-based vs. others), areas (nonurban vs. others), mean or median follow-up period (≥ 5 vs. < 5 years), year of study initiation (before the year 2000 vs. in the year 2000 or later), and sample size (≥ 50,000 vs. < 50,000). We computed p values for comparisons between subgroups using the χ^2^ test with one degree of freedom. To further explore potential sources of heterogeneity in the results, we conducted meta-regression analyses [24,25] with stratification according to year of study initiation (before the year 2000 vs. in the year 2000 or later). In the meta-regression analyses, we used the following characteristics as covariates: definition of incident diabetes (self-report vs. laboratory data), source of subjects (population-based vs. others), follow-up period (per 5-year increase), sample size (per 10,000 increase), and areas (provincial vs. others). All analyses were performed using Stata version 12.1 (StataCorp, College Station, TX).

## Results

### Literature Search

**Figure 1:**
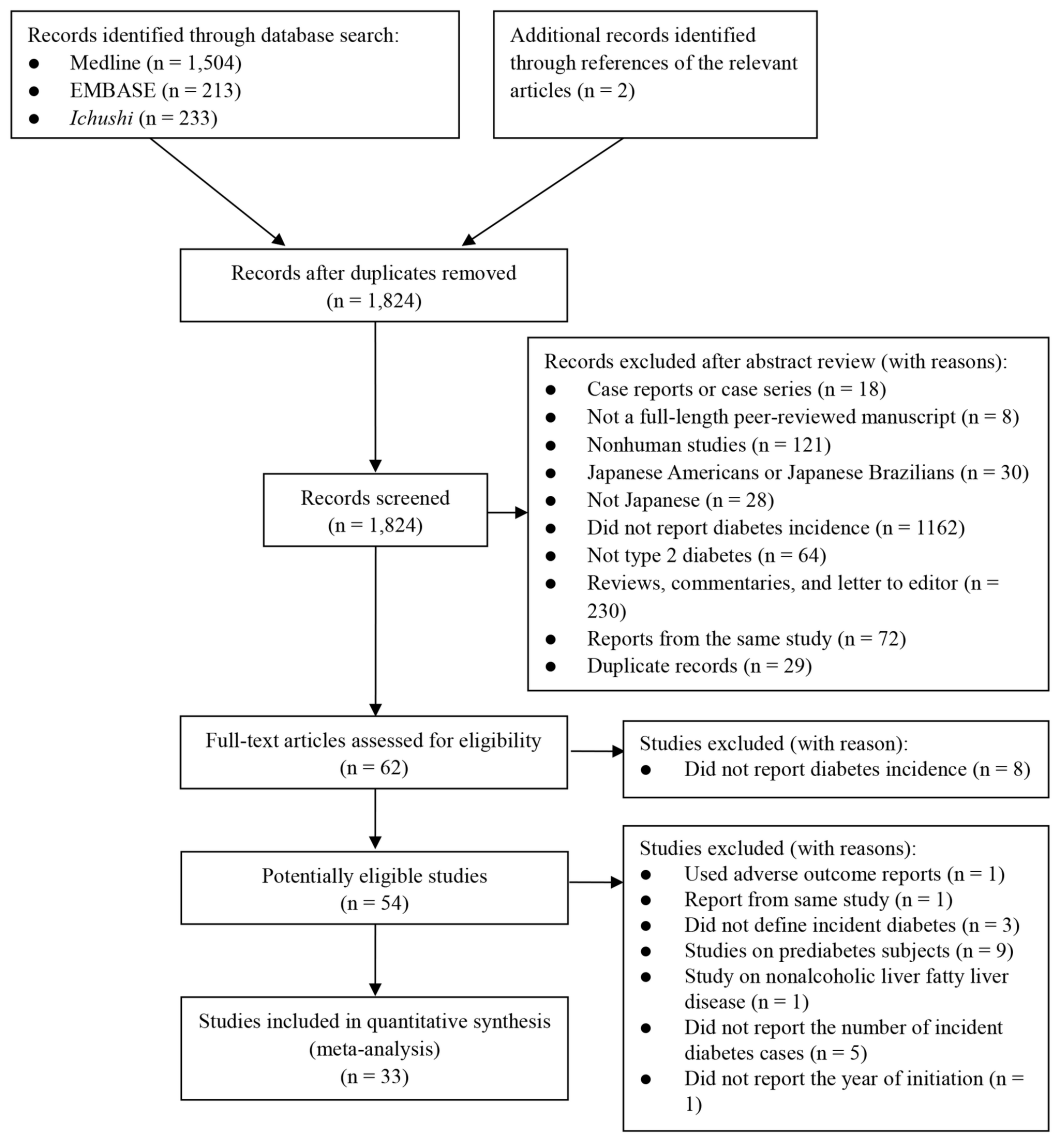
Literature search.

Initially, we identified 1824 related articles. Based on the titles and abstracts, 62 articles were considered potentially eligible, and the entire texts of these 62 articles were evaluated. After excluding 8 studies that did not report diabetes incidence, 54 relevant studies were further assessed for their eligibility (Figure 1). Of these 54 studies, 1 study based the ascertainment of incident type 2 diabetes on adverse outcome reports [26], 1 used an overlapping population [27], 3 did not define ascertainment of type 2 diabetes [28–30], 9 were studies on prediabetes populations [31–39], 1 was a study on nonalcoholic liver fatty liver disease patients (n = 1) [40], 2 did not report the follow-up period [41,42], 5 did not report the number of incident diabetes cases [41-45], and 1 did not report the year of study initiation [46]. All these studies were excluded, leaving 33 studies for the meta-analysis.

### Study Characteristics

The number of participants, year, and the definition of diabetes diagnosis used in the selected studies are summarized in Table 1 [14,15,47–76]. The follow-up period was 1–15 years, and participants were followed monthly up to 10 years. Three studies based the definition of incident type 2 diabetes on self-reports only [14,47,48], 10 on laboratory data only (fasting glucose levels, casual glucose levels, 2-h post-load glucose levels after oral glucose tolerance tests, or HbA1c levels) [15,49-57], and 20 on self-reports and laboratory data [50,58–76]. Nine studies were population-based studies [14,47,49,51,53,64,68,69,77] with participation rates varying from 40.9% to 85.3%. Six studies [48,49,51,53,68,69] were performed in nonurban areas.

**Table 1:** Characteristics of the studies included in the systematic review.

	Study	Year of study initiation	Sample size (men, %)	Source of subjects		Participation rate (%)^*^	Mean Age (range)	Follow-up, years	Definition of incident diabetes
**(i)**	**Laboratory data only**								
	Maegawa et al. [53]	1980	1,338 (42)	Population-based (The Aito Study, Aito Town, Shiga)		79.3	50.0 (40–64)	5.6	FPG ≥ 140 mg/dL, 2-h PG ≥ 200 mg/dL
	Tanabe et al.(1) [56]	1980	230 (70)	Health checkups (Nishikawa town, Niigata)		–	55.9 (≥20)	4.3	FPG ≥ 126 mg/dL, 2-hPG ≥ 200 mg/dL
	Taniguchi et al. [15]	1981	6,356 (100)	Health checkups (The Osaka Health Survey, Work site, Osaka)		–	41.5 (35–60)	9.7	FPG ≥ 126 mg/dL, 2-hPG ≥ 200 mg/dL
	Kawakami et al. [52]	1984	2,380 (100)	Health checkups (Work site, Japan)		–	N.A. (18–53)	8	FPG ≥ 140 mg/dL, 2-h PG ≥ 200 mg/dL
	Yoshinaga et al. [57]	1986	1,604 (80)	Health checkups (Single center, Tokyo)		–	51.2 (20–81)	4.5	FPG ≥ 120 mg/dL more than twice
	Nakano et al. [55]	1991	435 (75)	Health checkups (Fukushima city, Fukushima)		–	51.9 (31–76)	2.3	FPG ≥ 140 mg/dL, 2-h PG ≥ 200 mg/dL
	Nakanishi et al. (1) [54]	1994	1,257 (100)	Health checkups (Work site, Osaka)		–	46.7 (35–59)	5	FPG ≥ 126 mg/dL
	Kameda et al. [51]	1995	940 (43)	Population-based (The Funagata Study, Funagata Town, Yamagata)		40.9	58.2 (N.A.)	5	FPG ≥ 140 mg/dL, 2-h PG ≥ 200 mg/dL
	Doi et al. [49]	2002	2,164 (40)	Population-based (The Hisayama Study, Hisayama Town, Fukuoka)		77.0	58.6 (40–79)	6	FPG ≥ 126 mg/dL, 2-h PG ≥ 200 mg/dL
	Fujita et al.(1) [50]	2002	27,760 (26)	Health checkups (Kashiwa City, Chiba)		–	61.8 (40–79)	4	FPG ≥ 126 mg/dL, HbA1c ≥ 6.9% (52 mmol/mol)
(ii)	**Laboratory data and self-reports of diagnosis/treatment**								
	Sawada et al. [74]	1985	4,187 (men)		Health checkups (Work site, Tokyo)	–	32.0 (22–40)	14	FPG ≥ 126 mg/dL, 2-h PG ≥ 200 mg/dL, diabetes treatment
	Nagaya et al. [65]	1988	25,196 (67)		Health checkups (Single center, Gifu)	–	43.8 (30–59)	7.3	Fasting serum glucose ≥ 126 mg/dL, diabetes treatment
	Okada et al. [69]	1989	717 (38)		Population-based (Yaeyama district, Okinawa)	58.9	55.0 (30–89)	10	FPG ≥ 126 mg/dL, 2-h PG ≥ 200 mg/dL, HbA1c ≥ 6.9% (52 mmol/mol), diabetes treatment
	Sairenchi et al. [70]	1993	128,141 (31)		Health checkups (Ibaraki)	–	N.A. (40–79)	4.8	FPG ≥ 126 mg/dL, casual PG ≥ 200 mg/dL, diabetes treatment
	Fujita et al.(2) [50]	1994	35,579 (21)		Health checkups (Chiba City, Chiba)	–	56.3 (40–79)	10.2	FPG ≥ 126 mg/dL, casual PG ≥ 200 mg/dL, self-reports of diagnosis
	Nakanishi et al. (2) [66]	1994	3,260 (100)		Health checkups (Work site, Japan)	–	N.A. (35–59)	7	FPG ≥ 126 mg/dL, diabetes treatment
	Ohnishi et al. [68]	1994	827 (40)		Population-based (The Tanno and Sobetsu Study, towns of Tanno and Sobetsu, Hokaido)	N.A.	N.A. (40–64)	10	FPG ≥ 126 mg/dL, diabetes treatment
	Sanada et al. [72]	1994	1,554 (62)		Health checkups (2 centers, Fukushima)	–	50.4 (23–80)	10	FPG ≥ 126 mg/dL, 2-h PG ≥ 200 mg/dL, diabetes treatment
	Inoue et al. [61]	1995	449 (76)		Health checkups (Work site, Japan)	–	45.6 (23–65)	7	FPG ≥ 126 mg/dL, diabetes treatment, self-reports of diagnosis
	Heianza et al. [60]	1997	6,241 (75)		Health checkups (The TOPICS, Single center, Tokyo)	–	49.9 (24–82)	4.7	FPG ≥ 126 mg/dL, HbA1c ≥ 6.5% (48 mmol/mol)
	Fukui et al. [58]	1998	4,153 (59)		Health checkups (Single center, Kyoto)	–	48.2 (N.A.)	8.2	FPG ≥ 126 mg/dL, diabetes treatment
	Nomura et al. [67]	1998	9,322 (51)		Health checkups (Work site, Japan)	–	51.5 (19–69)	6	FPG ≥ 126 mg/dL, HbA1c ≥ 6.5%, diabetes treatment
	Tanabe et al.(2) [75]	1998	6,775 (32)		Health checkups (Tokachimachi City, Niigata)	–	62.0 (40–89)	5	FPG ≥126 mg/dL, casual PG ≥200 mg/dL, HbA1c ≥ 6.9% (52 mmol/mol), self-reports of diagnosis
	Hayashino et al. [59]	1999	4,975 (100)		Health checkups (The HIIPOP-OHP Study, Work site, Japan)	–	38.3 (19--69)	3.4	FPG ≥ 126 mg/dL, casual PG ≥ 200 mg/dL, diabetes treatment, self-reports of diagnosis
	Kato et al. [62]	2000	11,369 (29)		Health checkups (The Omiya MA Cohort Study, Omiya City, Saitama)	–	62 (55–68)	7	FPG ≥ 126 mg/dL, diabetes treatment, self-reports of diagnosis
	Sato et al. [73]	2000	10,631 (100)		Health checkups (The Kansai Healthcare Study, Work site, Kansai district)	–	47.9 (40–55)	4	FPG ≥ 126 mg/dL, diabetes treatment
	Muraki et al. [64]	2001	4,398 (36)		Population-based (The CIRCS, 5 areas, Japan)	N.A.	57.6 (40–69)	3	Fasting serum glucose ≥ 126 mg/dL, casual serum glucose ≥ 200 mg/dL, diabetes treatment
	Li et al. [63]	2002	3,008 (77)		Health checkups (Work site, Aichi)	–	47.3 (35–66)	6	Fasting glucose ≥ 126 mg/dL, self-reports of diagnosis
	Sakurai et al. [71]	2003	1,995 (100)		Health checkups (Work site, Toyama)	–	46.0 (35–55)	4.5	FPG ≥ 126 mg/dL, 2-h PG ≥ 200 mg/dL, diabetes treatment
	Totsuka et al. [76]	2003	172 (70)		Health checkups (Single center, Tsukuba City, Ibaraki)	–	49.4 (31–62)	3	FPG ≥ 126 mg/dL, 2-hPG ≥ 200 mg/dL, self-reports of diagnosis
**(iii)**	**Self-reports of diabetes diagnosis only**								
	Iso et al. [47]	1988	17,413 (39)		Population-based (The JACC Study, 45 areas, Japan)	83	53.2 (40–79)	5	Self-reports of diagnosis
	Kurotani et al. [14]	1995	48,437 (44)		Population-based (The JPHC Study, 11 areas, Japan)	81	50.7 (40–69)	5	Self-reports of diagnosis
	Oba et al. [48]	1992	13,540 (44)		Population-based (The Takayama Study, Takayama City, Gifu)	85.3	51.6 (≥35)	10	Self-reports of diagnosis

Abbreviations:

* Participation rates in population-based studies are shown.

### Incidence Rate of Type 2 Diabetes

**Figure 2:**
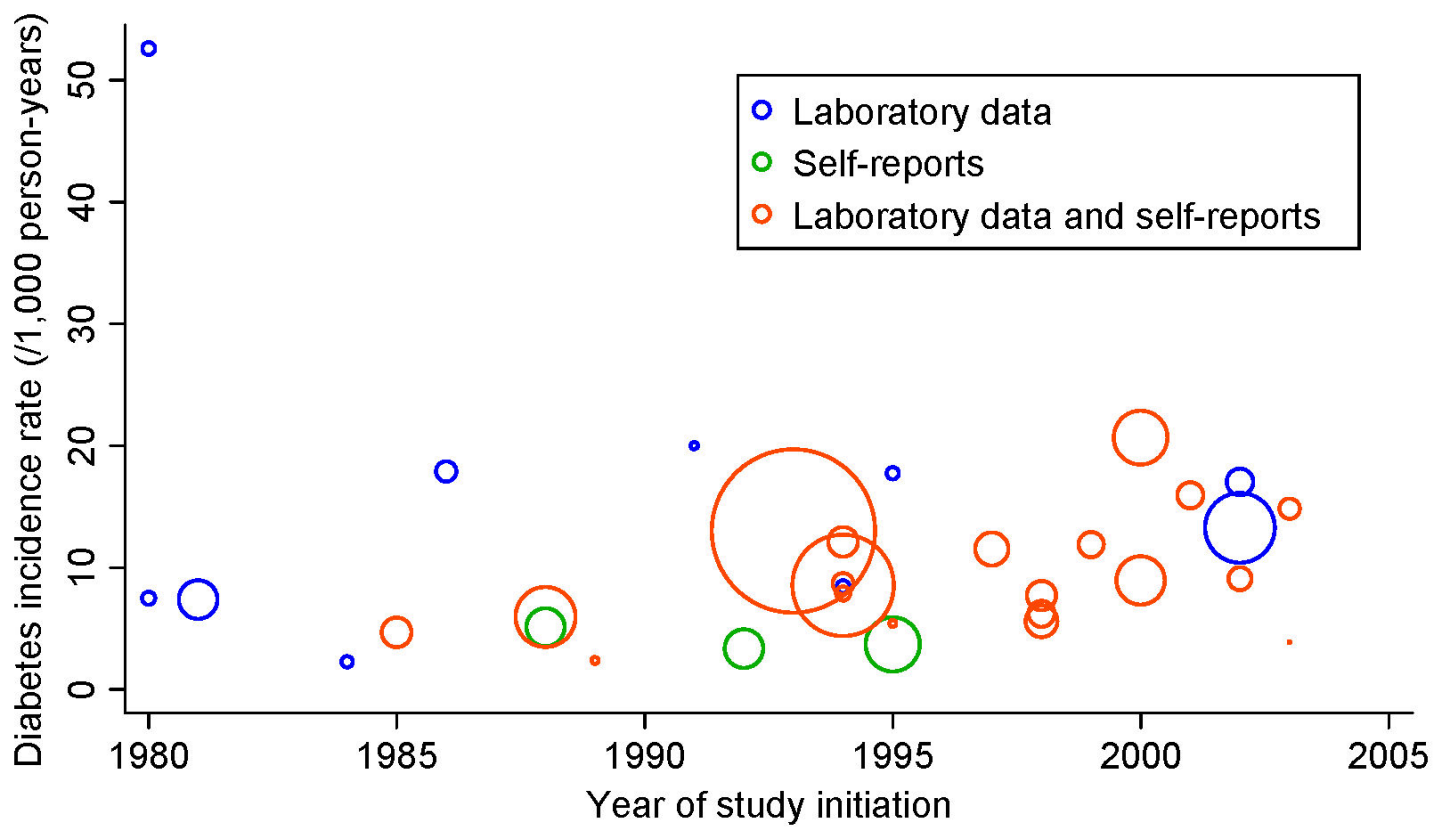
Forest plots of diabetes incidence rate. CI indicates confidence interval.Dots indicate diabetes incidence rates. Horizontal lines indicate 95% CIs for incidence rates. The diamonds represent the pooled incidence rate estimates with 95% CIs.

**Figure 3:**
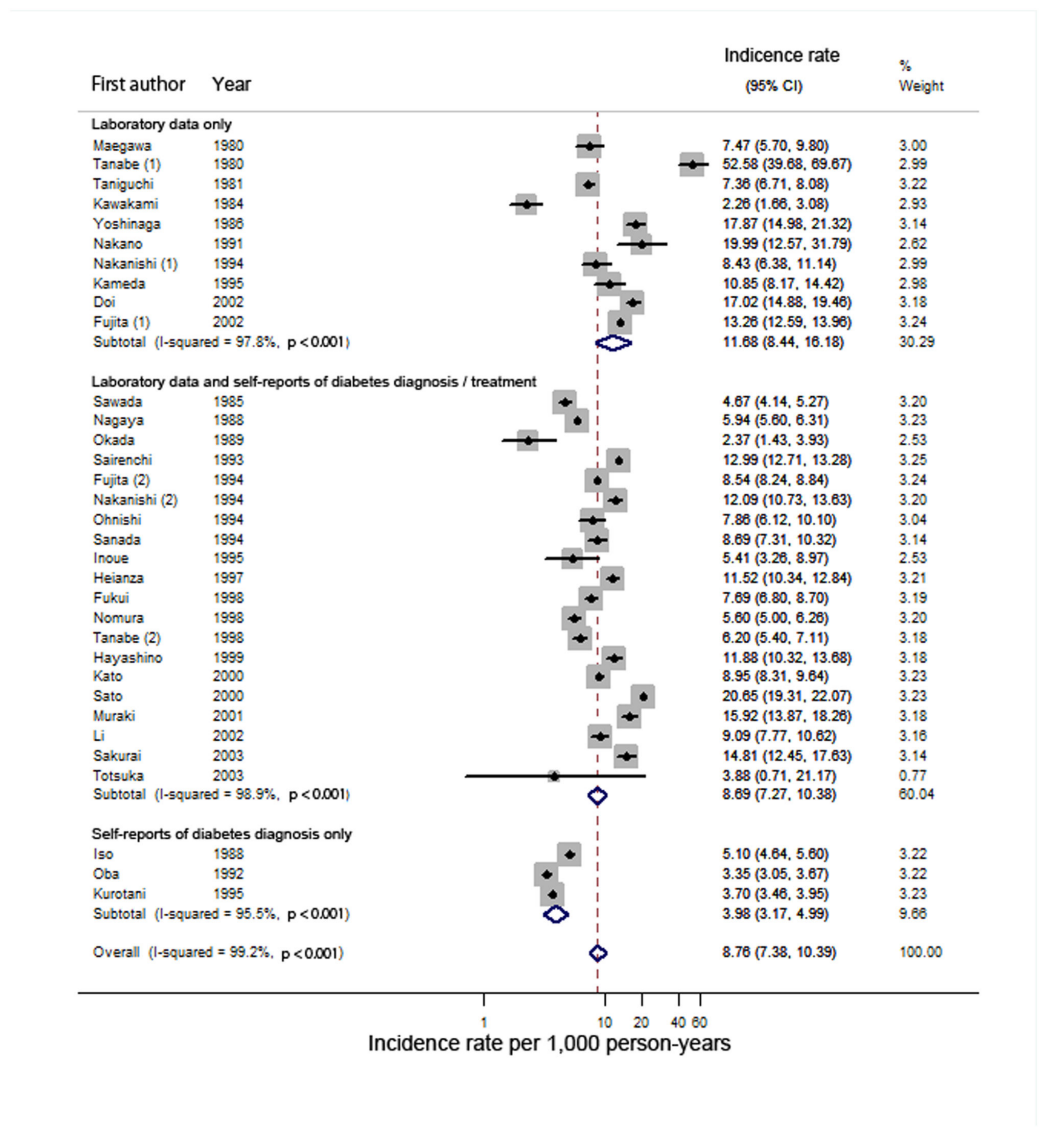
Bubble plots of diabetes incidence rate against the year of study initiation. A bubble shows a study, and the size of the bubble is proportional to the inverse of the variance of the log-transformed incidence rate. Diabetes incidence rate was calculated by dividing the number of new-onset diabetes cases by the duration of follow-up. When the mean follow-up duration was not available, the median was used.

The 33 studies included 386,803 participants. The random-effects model indicated that the pooled incidence rate of diabetes was 8.8 (95% CI = 7.4–10.4) per 1,000 person-years (Figure 2). There was little evidence of publication bias. The funnel plot did not indicate asymmetry; Begg’s p value was 0.45; and Egger’s bias coefficient was -3.98 (95% CI, -9.72-1.77; p = 0.17) (not shown). We observed a high degree of heterogeneity (I^2^ = 99.2%; p < 0.001), with incidence rates ranging from 2.3 to 52.6 per 1000 person-years. We also performed stratified analyses according to the definition of incident diabetes (self-reports vs. laboratory data), source of subjects (population-based vs. others), areas (nonurban vs. others), mean or median follow-up period (≥ 5 vs. < 5 years), year of study initiation (before the year 2000 vs. in the year 2000 or later 2000), and sample size (≥ 50,000 vs. < 50,000) (Table 2). The studies using self-reports of diabetes alone for diabetes diagnosis showed a lower diabetes incidence rate (N of studies = 3; pooled incidence rate = 4.0; 95% confidence interval = 3.2–5.0; p for interaction < 0.001) than did the studies using laboratory data (N of studies = 30; pooled incidence rate = 9.6; 95% CI = 8.3–11.1). The studies with longer follow-up periods (≥5 years) showed lower incidence rate estimates of diabetes (N of studies = 22; pooled incidence rate = 6.6; 95% CI = 5.5–8.0; p for interaction < 0.001) than did the studies with shorter follow-up periods (< 5 years; N of studies = 11; pooled incidence rate = 16.3, 95% CI = 14.0–18.9). The studies that initiated before the year 2000 (N of studies = 25) reported lower estimates of incidence rates (pooled incidence rate = 7.8; 95% CI = 6.2–9.5; p for interaction = 0.001) than did the studies that initiated in the year 2000 or later (N of studies= 8; pooled incidence rate = 13.4; 95% CI = 10.4–17.1). Figure 3 shows a bubble plot of the diabetes incidence rate per 1,000 person-years as a function of the year of study initiation. The results indicated that more recent studies tended to show higher incidence rate estimates. However, stratification according to these characteristics could not entirely explain the heterogeneity in the results, with I^2^ statistics being high within each stratum. We also conducted meta-regression analyses to further explore the sources of heterogeneity (Table 3). Meta-regression analyses indicated that a longer follow-up period was associated with lower incidence rates in studies before the year 2000; however, it explained only a small proportion of the heterogeneity (adjusted R^2^ statistics = 22.1%; residual I^2^ statistics = 99.1%). In addition, we estimated the pooled incidence rate of diabetes in the studies on prediabetes populations. The incidence rate among prediabetes populations (pooled incidence rate = 49.2 per 1,000 person-years; 95% CI = 31.5–76.8) (not shown) [31,32,34–39] was much higher than that among total populations (pooled incidence rate = 8.8 per 1,000 person-years).

**Table 2:** Stratified analysis of the incidence rate of diabetes.

**Group**	**Number of studies**	Incidence rate^*^ (95% CI)	**p value** (heterogeneity^†^)	**I^2^ (%)**	**p value** (interaction^‡^)
Total	33	8.8 (7.4–10.4)	< 0.001	99.2	
Definition of incident diabetes					< 0.001
Laboratory data	30	9.6 (8.3–11.1)	< 0.001	97.6	
Self-reports only	3	4.0 (3.2–5.0)	< 0.001	95.5	
Source of subjects					0.13
Population-based	9	6.7 (4.3–10.4)	< 0.001	99.0	
Others	24	9.7 (8.2–11.4)	< 0.001	98.9	
Area					0.40
Nonurban	6	6.7 (3.3–13.7)	< 0.001	98.8	
Others	27	9.2 (7.7–11.1)	< 0.001	99.2	
Follow-up period					< 0.001
≥5 years	22	6.6 (5.5–8.0)	< 0.001	98.3	
<5 years	11	16.3 (14.0–18.9)	< 0.001	96.5	
Year of study initiation					0.001
≥ 2000	8	13.4 (10.4–17.1)	< 0.001	97.8	
< 2000	25	7.8 (6.3–9.5)	< 0.001	99.2	
Sample size					0.39
≥ 10,000	9	7.8 (5.6–10.8)	< 0.001	99.7	
< 10,000	24	9.2 (7.5–11.3)	< 0.001	97.2	

Abbreviation:

* Incidence rate estimates were obtained using a random-effects model.

† p values for heterogeneity across studies were computed using Cochrane’s Q test.

‡ p values for comparisons between subgroups were computed using the χ^2^ test with one degree of freedom.

**Table 3:** Meta-regression analyses of the incidence rate of diabetes with stratification according to year of study initiation (before the year 2000 vs. in the year 2000 or later).

**Study characteristic**	Ratio of incidence rate^*^ (95% CI)	**p value**	**Adjusted R^2^**	**Residual I^2^ (%)**
**Studies before the year 2000 (N = 25)**				
Self-reports only	0.47 (0.21–1.04)	0.06	12.4	98.6
Population-based	0.57 (0.32–1.03)	0.06	11.3	98.7
Nonurban areas	0.66 (0.33–1.33)	0.24	1.7	99.2
5-year increase in follow-up period	0.55 (0.35–0.86)	0.01	22.1	99.1
5-year increase in year of study initiation	0.96 (0.75–1.23)	0.73	-4.1	99.3
10,000 increase in sample size	1.00 (0.90–1.12)	0.94	-4.8	98.7
**Studies in the year 2000 or later (N = 8)**				
Population-based	1.33 (0.67–2.64)	0.35	-1.4	98.0
Nonurban areas	1.32 (0.52–3.34)	0.49	-9.5	98.1
5-year increase in follow-up period	0.54 (0.19–1.51)	0.19	31.1	96.3
5-year increase in year of study initiation	0.82 (0.17–3.96)	0.76	-21.4	98.1
10,000 increase in sample size	1.00 (0.68–1.49)	0.98	-21.4	98.1

Abbreviation:

* Incidence rate with characteristic divided by incidence rate without characteristic. Ratios < 1 correspond to a smaller incidence rate for studies with the characteristic.

### Validity of Self-reported Diabetes

Among the studies that considered self-reports for the definition of diabetes diagnosis, 3 conducted validity studies among participants whose laboratory data were available [14,47,63]. In the Japan Collaborative Cohort Study for Evaluation of Cancer Risk Study (JACC Study), self-reports were compared with laboratory data and treatment status in a subsample of study participants [47]. In the Japan Public Health Center-based prospective Study (JPHC Study) [14], self-reports were compared with medical records and laboratory data retrieved from health checkups [78,79]. In the study by Li et al [63], self-reports were compared with laboratory data and reports from the physicians of study participants [80]. Their positive predictive values, negative predictive values, sensitivity, and specificity were 95.7%-99.2%, 93.8%–96.3%, 70%-82.6%, and 95%–99.7%, respectively [47,78,80]. Because these validation studies were conducted among participants whose laboratory data were available, validity of self-reports among those who had not visited health checkups remains unclear.

## Discussion

In the present systematic review and meta-analysis of studies that evaluated new-onset type 2 diabetes in the Japanese population, we found that there was a high degree of heterogeneity in the incidence of diabetes in Japan and an increasing number recent studies tended to show higher incidence rate estimates. Our study also indicated that studies that used self-reported diagnosed diabetes tended to show a lower incidence rate than studies that used laboratory data, suggesting that laboratory data are important for the accurate estimation of the incidence rate of diabetes. In addition, the studies with longer follow-up durations tended to show lower incidence rates. In the cohorts with longer follow-up durations, individuals who did not develop diabetes at earlier stages of study period were likely less predisposed toward diabetes and would have had a lower likelihood of developing diabetes later in the study, which might have led to the lower overall incidence rates in the studies with follow-up durations that were longer than those of the others. Although we observed a high degree of heterogeneity between studies, stratified analyses or meta-regression analyses did not identify major sources of the heterogeneity.

The overall incidence rate of diabetes in Japan was found to be 9.0 per 1,000 person-years. This estimate is slightly higher than the self-report-based [81,82] or administrative database-based [83] estimates from the U.S. [81], U.K. [83], and China [82]. The U.S. National Health Interview Survey reported that the incidence rate of medically diagnosed diabetes was 8.4 per 1,000 person-years among men and 8.1 per 1,000 person-years among women in 2008 [81]. Using a primary care medical records database in the U.K, the incidence rate of diabetes in the U.K. was reported to be 4.4 per 1,000 person-years in 2005 [83]. In addition, the Shanghai Diabetes Study reported that diabetes incidence rate identified by self-reports was 6.0 per 1,000 person-years among Chinese women in Shanghai [82]. However, because estimates based on self-reports or administrative databases would have overlooked undiagnosed or untreated diabetes, these studies may have underestimated the incidence rate. Indeed, our overall estimate of diabetes incidence in Japan was mainly driven by the incidence rates from studies using laboratory data. The overall rate (9.0 per 1,000 person-years) was close to that observed in the study among Australians, in which diabetes was defined by fasting plasma glucose levels ≥126 mg/dL and/or diabetes diagnosed by physicians [84]. In the Blue Mountains Eye Study, the incidence rate of type 2 diabetes was 9.3 per 1,000 person-years among non-Aboriginal Australians [84]. Further studies that standardize the definition of incident diabetes are required to compare the incidence rate of diabetes between countries.

Diabetes is often defined exclusively on the basis of self-reports [85,86]. In the present review, we found that studies based on self-reports alone tended to show a lower incidence rate compared with studies based on laboratory data, suggesting that laboratory data are important to estimate the incidence rate of diabetes correctly. Three studies conducted validation studies among participants whose laboratory data were available; the range for the specificity of self-reports as obtained in this review (95–99.7%) was relatively high. In studies based on self-reports, diabetes incidence may have been underestimated probably because the sensitivity was not sufficiently high. Moreover, the validity of self-reports among those who had not visited health checkups is unclear. In particular, the sensitivity of self-reports among participants who had not been screened for diabetes may be much lower than the range (70%–82.6%) obtained in this review. Of note, laboratory data were not available in any of the large-scale population-based studies [14,47,77]. This seems to indicate that multiple sources of evidence including self-reports, claim-based data, hospital admission data, and mortality data should be considered in such situations.

Our study also indicated that the incidence of type 2 diabetes in Japan may be increasing. The FPG threshold was lowered from ≥140 to ≥126 mg/dl by the ADA, WHO, and JDS in 1997, 1998, and 1999, respectively [6,8,9]; this may have reflected the change in the diagnoses and incidence rates of diabetes. The increase in obesity prevalence [87], decline in physical activity [5], and population aging [88] may also explain possible trend toward an increasing rate of diabetes incidence in Japan. Future studies using the standardized definition of incident type 2 diabetes are warranted to clarify the trend in the incidence of diabetes in Japan.

The strengths of this study include its large sample size and comprehensive assessment of definitions used to identify incident type 2 diabetes. Several limitations also exist. First, we limited our search to the Japanese population, which limits the generalizability our findings. Second, we did not have individual participant data or age- and gender-specific estimates of type 2 diabetes incidence. Therefore, we were not able to compute age-standardized incidence rates. Third, although we searched 3 large electronic databases (MEDLINE, EMBASE, and *Ichushi* [the largest database for medical literature in Japan]), we may have missed some related studies. Finally, large regional differences in diabetes incidence may exist, but we were unable to establish a region-specific estimate.

## Conclusions

Our systematic review and meta-analysis indicated the presence of a high degree of heterogeneity, which suggests that there is a considerable amount of uncertainty regarding the incidence of type 2 diabetes in Japan. They also suggested that laboratory data may be important to identify undiagnosed diabetes. Future studies should aim to standardize the definition of incident diabetes in order to compare the incidence rate of type 2 diabetes between countries

## Supporting Information

Checklist S1 (DOCX)
