# Correction: Incidence of Type 2 Diabetes in Japan: A Systematic Review and Meta-Analysis

**DOI:** 10.1371/annotation/c9e78482-03d2-4cc3-b971-7d91f6ac6cab

**Published:** 2013-10-21

**Authors:** Atsushi Goto, Maki Goto, Mitsuhiko Noda, Shoichiro Tsugane

Figures 2 and 3 were incorrectly switched. The image currently appearing as Figure 2 belongs with the title and legend of Figure 3, and the image currently appearing as Figure 3 belongs with the title and legend of Figure 2. The titles and legends are in correct order. 

